# Long-Term Outcomes of Transcatheter vs Surgical Aortic Valve Replacement: Meta-analysis of Randomized Trials

**DOI:** 10.1016/j.jscai.2024.102143

**Published:** 2024-05-15

**Authors:** Giuseppe Talanas, Angelo Laconi, Dean J. Kereiakes, Pierluigi Merella, Michael J. Reardon, Andrea Spano, Gerardo Petretto, Francesco Lauriola, Marta Casula, Valentina Micheluzzi, Mehriban Isgender, Yiannis Chatzizisis, Michael E. Farkouh, Alexandra J. Lansky, Nicolo Piazza, Michele Portoghese, Gavino Casu, Eliano Pio Navarese

**Affiliations:** a Clinical Experimental Cardiology, Clinical and Interventional Cardiology, University of Sassari, Sassari, Italy; b SIRIO MEDICINE Research Network, Sassari, Italy; c The Christ Hospital and Lindner Research Center, Cincinnati, Ohio; d Department of Cardiovascular Surgery, Houston Methodist Hospital, Houston, Texas; e Republican Clinical Hospital, Department of Cardiology, Azerbaijan Medical University, Department of Family Medicine, Baku, Azerbaijan; f Center for Digital Cardiovascular Innovations, Division of Cardiovascular Medicine, Miller School of Medicine, University of Miami, Miami, Florida; g Cedars-Sinai Health System, Los Angeles, California; h Section of Cardiovascular Medicine, Department of Internal Medicine, Yale School of Medicine, New Haven, Connecticut; i McGill University Health Center, Montreal, Quebec, Canada; j Department of Cardiovascular Surgery, University of Sassari, Sassari, Italy

**Keywords:** meta-analysis, randomized trials, surgical aortic valve replacement, transcatheter aortic valve replacement

## Abstract

**Background:**

We aimed to perform a meta-analysis of randomized trials comparing long-term outcomes of patients undergoing transcatheter aortic valve replacement (TAVR) vs surgical aortic valve replacement (SAVR) for severe aortic stenosis. The short-term efficacy and safety of TAVR are proven, but long-term outcomes are unclear.

**Methods:**

We included randomized controlled trials comparing TAVR vs SAVR at the longest available follow-up. The primary end point was death or disabling stroke. Secondary end points were all-cause mortality, cardiac mortality, stroke, pacemaker implantation, valve thrombosis, valve gradients, and moderate-to-severe paravalvular leaks. The study is registered with PROSPERO (CRD42023481856).

**Results:**

Seven trials (N = 7785 patients) were included. Weighted mean trial follow-up was 5.76 ± 0.073 years. Overall, no significant difference in death or disabling stroke was observed with TAVR vs SAVR (HR, 1.02; 95% CI, 0.93-1.11; *P* = .70). Mortality risks were similar. TAVR resulted in higher pacemaker implantation and moderate-to-severe paravalvular leaks compared to SAVR. Results were consistent across different surgical risk profiles. As compared to SAVR, self-expanding TAVR had lower death or stroke risk (*P* interaction = .06), valve thrombosis (*P* interaction = .06), and valve gradients (*P* interaction < .01) but higher pacemaker implantation rates than balloon-expandable TAVR (*P* interaction < .01).

**Conclusions:**

In severe aortic stenosis, the long-term mortality or disabling stroke risk of TAVR is similar to SAVR, but with higher risk of pacemaker implantation, especially with self-expanding valves. As compared with SAVR, the relative reduction in death or stroke risk and valve thrombosis was greater with self-expanding than with balloon-expandable valves.

## Introduction

Symptomatic severe aortic stenosis (AS) is associated with poor prognosis. Historically, surgical aortic valve replacement (SAVR) has been the definitive treatment for severe AS and has been associated with improved survival rates and enhanced quality of life.^1^ Transcatheter aortic valve replacement (TAVR) has emerged as a less invasive therapeutic alternative for patients with increased surgical risk, expanding treatment options for patients with severe AS.^2^ TAVR has demonstrated promising short-term to intermediate-term outcomes in terms of both efficacy and safety, leading to its consideration for a wider range of patients, including those with lower surgical risk.^3^^,^^4^ However, the long-term performance and durability of TAVR relative to SAVR remain to be conclusively determined.^5^^,^^6^ Recently, results from trials with longer follow-up periods have become available.^7^, ^8^, ^9^, ^10^ As TAVR has become the dominant strategy for severe AS in patients aged 65 years and older (>90%),^11^ the assessment of long-term valve outcomes becomes fundamental to its broader application.

Within this framework, we aimed to perform a meta-analysis of randomized trials investigating long-term clinical outcomes of TAVR vs SAVR.^11^

## Methods

The current study adhered to the established guidelines for systematic reviews and meta-analyses, as outlined in the published Preferred Reporting Items for Systematic Reviews and Meta-Analyses recommendations.^12^ The current meta-analysis is registered in the International Prospective Register of Systematic Reviews (PROSPERO) under the registration number CRD42023481856. The study is a meta-analysis of published randomized trials, which does not require ethical approval.

### Search strategy

We conducted a systematic search of relevant medical databases, including the Cochrane Central Register of Controlled Trials, MEDLINE and Embase, and Google Scholar, covering the period from December 2000 to November 2023. The search criteria encompassed trials comparing TAVR and SAVR for patients with severe AS. Search queries included “severe aortic stenosis” or “severe symptomatic aortic stenosis,” “TAVR” or “transcatheter aortic valve replacement,” and “SAVR” or “aortic valve replacement.” In addition to electronic searches, we manually examined the references of selected studies and meta-analyses to identify any additional eligible studies.

Backward snowballing was performed and abstracts from major congress proceedings were searched. Published meta-analyses on the subject were screened, and the data were critically appraised and cross-checked with the original studies. To ensure comprehensive coverage, we also searched conference abstracts from medical proceedings, including the American Heart Association, the American College of Cardiology, the European Society of Cardiology, Transcatheter Therapeutics, Transcatheter Valve Therapies, and EuroPCR. Supplemental Table S1 outlines the full electronic MEDLINE search process.

### Inclusion criteria

Trials were considered eligible for inclusion if they provided clinical outcome data of interest comparing TAVR vs SAVR at the longest available follow-up period. The minimum trial follow-up duration should exceed 1 year. Observational studies were not included in the analysis.

### End points

The primary end point of our analysis is the composite outcome of death or disabling stroke, using data obtained from the individual trials. We also explored secondary end points, which encompassed all-cause mortality, mortality related to cardiovascular (CV) causes, the incidence of stroke, the implantation of new permanent pacemakers, valve thrombosis, valve gradients, and the presence of moderate/severe paravalvular leaks. We adhered to the definitions provided by each trial for each adverse event.

### Data extraction and data analysis

In our analysis, we utilized the Cochrane Risk of Bias 2.0 tool to assess the included studies. We conducted outcomes analysis on an intention-to-treat basis. For clinical outcomes, we pooled hazard ratios (HR), along with their 95% CI which account for time-to-event data and follow-up durations. If HR were not consistently available from original trials; risk ratios (RR) were utilized instead. Valve gradients were compared using mean values, expressed as mean ± SD. The longest available follow-up data beyond 1 year were abstracted. For the meta-analyses, pooled HR were calculated using the DerSimonian and Laird random-effects model. Interaction testing was performed for risk and TAVR group comparisons. Heterogeneity was assessed using the I^2^ statistic, with defined levels of low, moderate, and significant heterogeneity. Potential publication bias was estimated visually and by linear regression.^13^

Trials were categorized into 3 groups based on surgical risk: high risk, intermediate risk, and low risk. The TAVR arm was further stratified into self-expanding and balloon-expandable groups. Significance testing was conducted at a 2-tailed 5% significance level. For interaction P values, a value of <.10 was considered statistically significant, as conventionally established.^14^ All statistical analyses were performed using the R programming environment.

## Results

### Study selection and patient population

**Figure 1:**
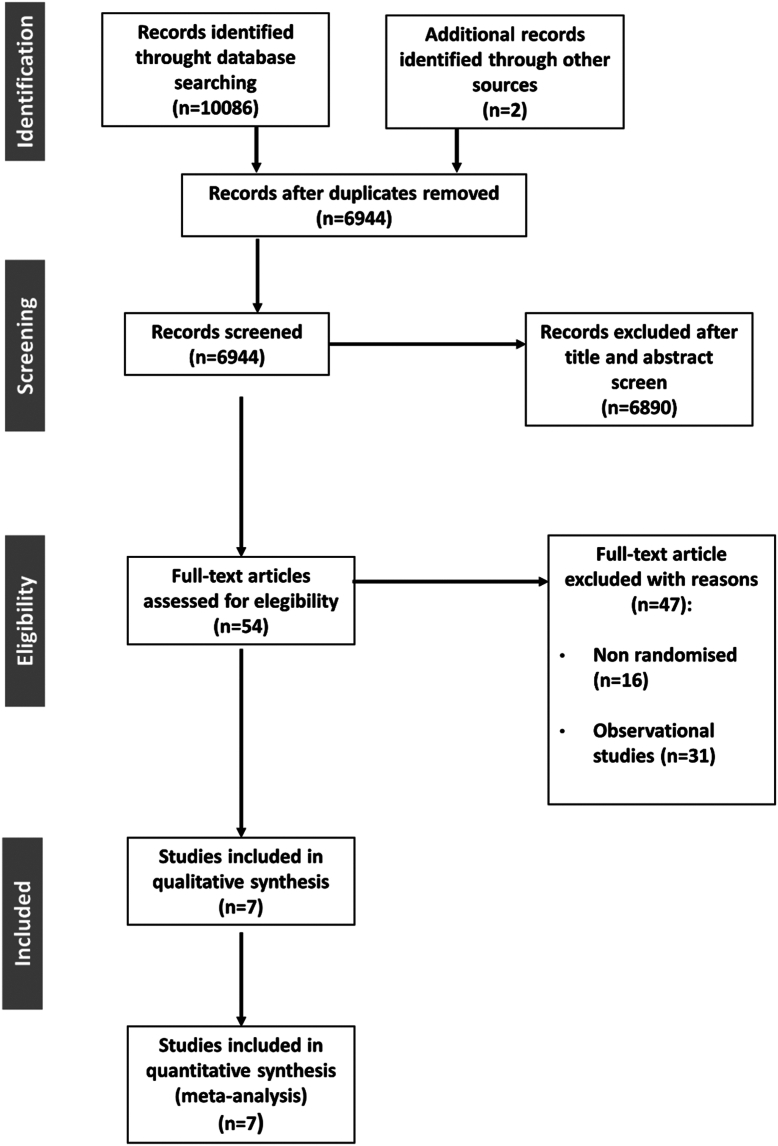
**Study selection.** TAVR, transcatheter aortic valve replacement; SAVR, surgical aortic valve replacement.

Seven trials involving a total of 7785 patients randomly allocated to TAVR (n = 3985) or SAVR (n = 3800) were included.^8^, ^9^, ^10^^,^^15^, ^16^, ^17^, ^18^, ^19^, ^20^, ^21^ Weighted mean trial follow-up was 5.76 ± 0.073 years. The Preferred Reporting Items for Systematic Reviews and Meta-Analyses flow diagram is shown in Figure 1. Key characteristics of included trials are reported in Supplemental Table S2. Valve thrombosis definitions are reported in Supplemental Table S3. The risk of bias assessment is reported in Supplemental Table S4. Overall, 6 trials were at low risk of bias in all domains, and 1 presented some concerns (Supplemental Table S4). There was no publication bias, with no significant Egger test for the explored outcomes (Supplemental Table S5).

We classified patients into 3 groups based on surgical risk—low, intermediate, and high—following the trial's classification criteria. In the TAVR group, which included 3985 patients, the distribution was as follows: 1371 were categorized as low risk, 1875 as intermediate risk, and 739 as high risk. In comparison, the SAVR group, consisting of 3800 patients, had 1273 low-risk, 1817 intermediate-risk, and 710 high-risk patients.

### Death or disabling stroke

**Figure 2:**
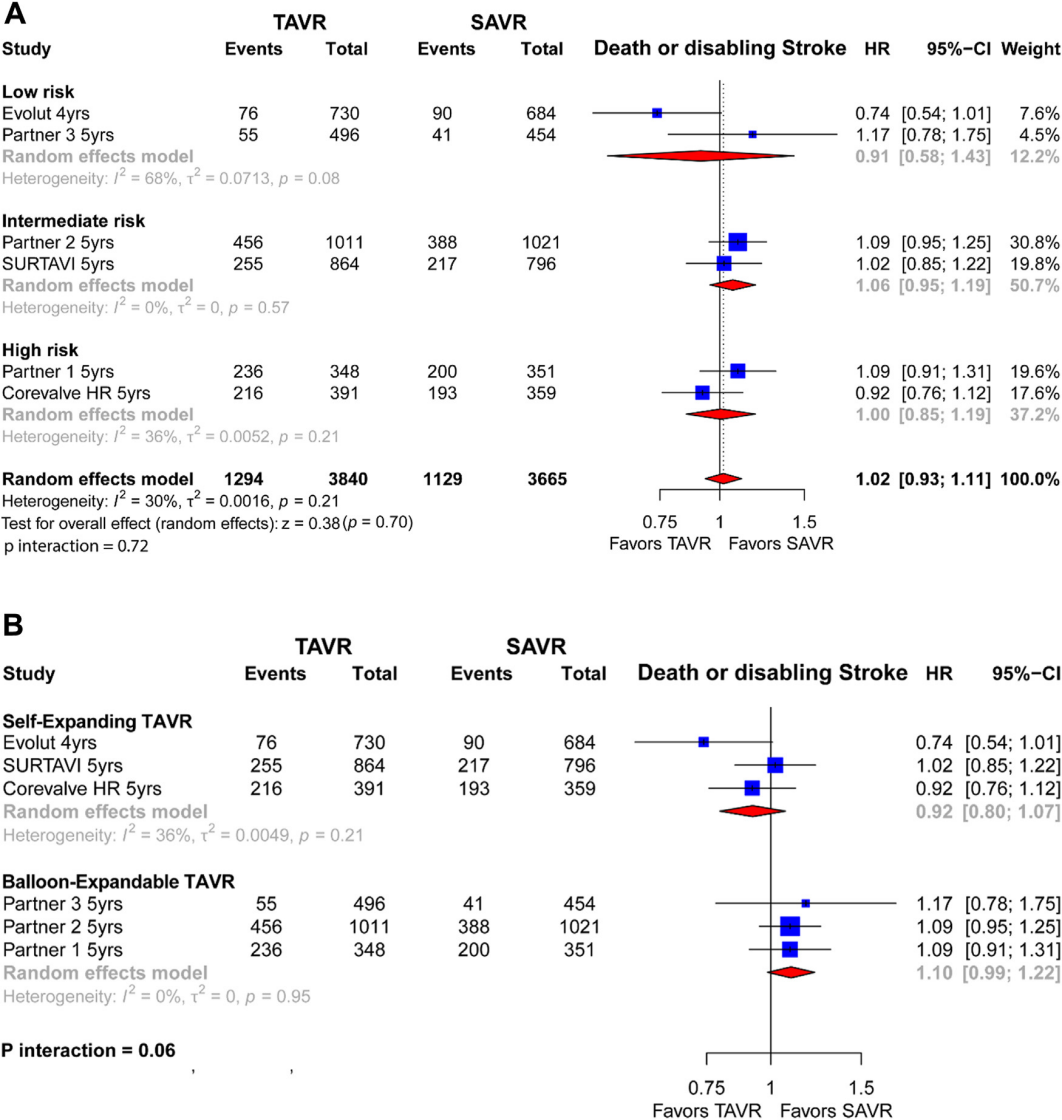
**Individual and summary hazard ratios (HR) with their CIs for mortality or disabling stroke of studies comparing transcatheter aortic valve replacement (TAVR) vs surgical aortic valve replacement (SAVR).** Results are stratified by surgical risk (**A**) and TAVR valve type (**B**).

A total of 6 trials including 7505 patients contributed to the death or disabling stroke outcome. Overall, 1294 of 3840 patients (33.7%) randomized to elective TAVR vs 1129 of 3665 patients (30.8%) randomized to SAVR experienced a death or stroke event without significant differences between strategies (HR, 1.02; 95% CI, 0.93-1.11; *P* = .70) (Figure 2A), with moderate statistical heterogeneity observed (I^2^ = 30%). Results remained consistent, regardless of the surgical risk (*P* interaction = .72).

In the analysis by transcatheter aortic valve type, when compared to SAVR, self-expanding TAVR yielded a lower risk of death or disabling stroke (27.55% vs 27.18%; HR, 0.92 [0.80-1.07]), than balloon-expandable TAVR (40.26% vs 34.44%; HR, 1.10 [0.99-1.22]; *P* interaction = .06) (Figure 2B).

**Figure 3:**
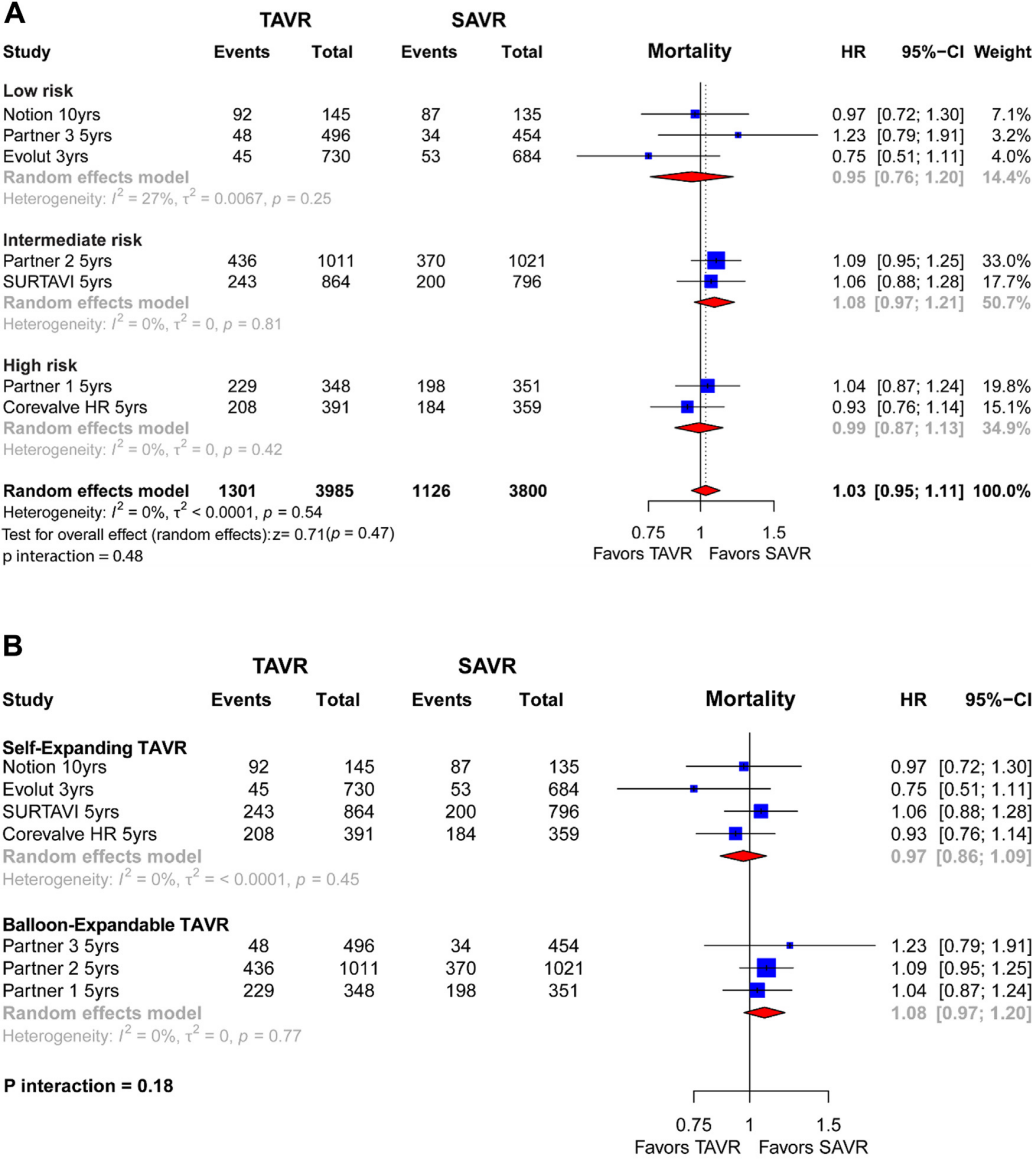
**Individual and summary hazard ratios (HR) with their CI for mortality of studies comparing transcatheter aortic valve replacement (TAVR) vs surgical aortic valve replacement (SAVR).** Results are stratified by surgical risk (**A**) and TAVR valve type (**B**).

A total of 7 trials including 7785 patients contributed to the all-cause mortality outcome. Overall, 1301 of 3985 patients (32.6%) randomized to elective TAVR vs 1126 of 3800 patients (29.6%) randomized to SAVR died of any cause. There was moderate statistical heterogeneity (I^2^ = 27%). Overall, there were no significant differences in all-cause mortality (HR, 1.03; 95% CI, 0.95-1.11; *P* = .54) (Figure 3A). Variations in surgical risk profiles did not affect results (*P* interaction = .48). In the analysis by transcatheter aortic valve type, as compared to SAVR, self-expanding TAVR yielded a comparable risk of death (27.60% vs 26.54%; HR, 0.97 [0.86-1.09]) to balloon-expandable TAVR (38,43% vs 32,96%; HR, 1.08 [0.97-1.20]; *P* interaction = .18) (Figure 3B).

Results for CV mortality were consistent with those of all-cause death. A total of 6 trials including 6031 patients contributed to CV mortality. Overall, 685 of 3104 patients (22.07%) randomized to elective TAVR vs 572 of 2927 patients (19.54%) randomized to SAVR died of cardiac death (HR, 0.99 [0.88-1.11]; *P* = .82). Heterogeneity was absent, as indicated by an I^2^ of 0% (Supplemental Figure S1**)**. The stratified analysis by TAVR type did not show meaningful differences in CV mortality in terms of differential effect of the self-expanding and balloon-expandable TAVR as compared to SAVR (Supplemental Figure S2).

A total of 7 trials including 7785 patients contributed to the any stroke outcome.

Overall, 391 of 3985 patients (9.81%) randomized to elective TAVR vs 370 of 3800 patients (9.74%) randomized to SAVR suffered from a stroke event.

Overall, there were no significant differences in any stroke (HR, 0.96 [0.81-1.13]; *P* = .41) (Supplemental Figure S3). Analyses by surgical risk did not show meaningful differences across distinct risk profiles (*P* interaction = .86).

In the analysis stratified by valve type, in comparison to SAVR, the self-expanding TAVR had a numerically lower stroke risk (HR, 0.86 [0.71-1.05]). In the balloon-expandable TAVR group, the HR was 1.10 (0.89-1.36), without significant differences between valve types (P interaction = 0.10) (Supplemental Figure S4).

### Pacemaker implantation

A total of 7 trials including 7785 patients contributed to the pacemaker implantation outcome.

Overall, 847 of 3985 patients (21.3%) randomized to elective TAVR vs 402 of 3800 patients (10.6%) randomized to SAVR had a pacemaker implant.

**Figure 4:**
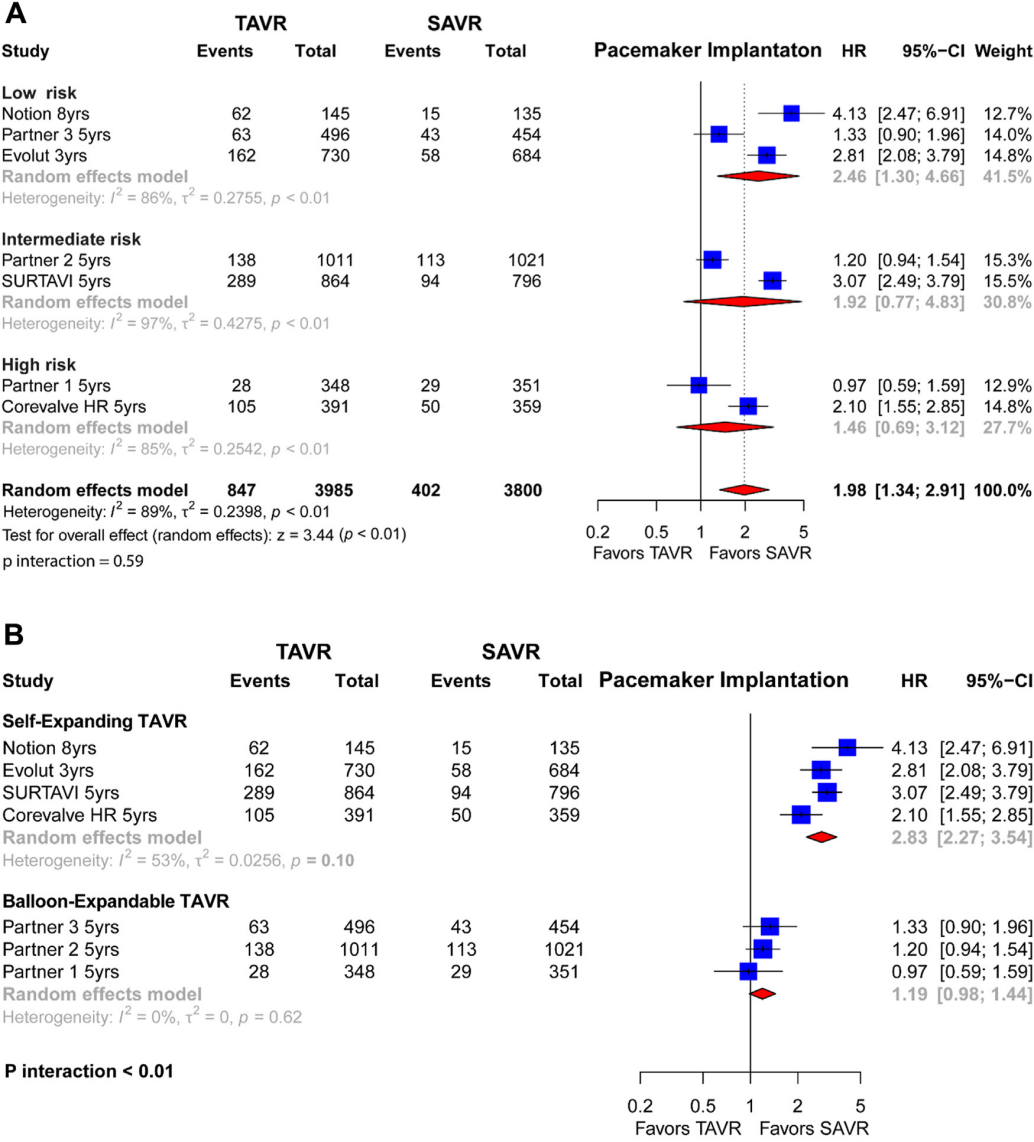
**Individual and summary hazard ratios (HR) with their CIs for pacemaker implantation of studies comparing transcatheter aortic valve replacement (TAVR) vs surgical aortic valve replacement (SAVR).** Results are stratified by surgical risk (**A**) and TAVR valve type (**B**).

Overall, TAVR carried a significantly higher risk of pacemaker implantation as compared to SAVR (HR, 1.98; 95% CI, 1.34-2.91; *P* < .01] (Figure 4A). Results were consistent across low-to-high surgical risk profiles, without significant differences (*P* interaction = .59).

Additionally, by stratification based on transcatheter valve type, as compared to SAVR, self-expanding TAVR yielded higher rates of pacemaker implantation (29.01% vs 11%; HR, 2.83 [2.27-3.54]), than balloon-expandable TAVR (12.34% vs 10.13%; HR, 1.19 [0.98-1.44]; *P* interaction < .01) (Figure 4B).

### Valve thrombosis

**Figure 5:**
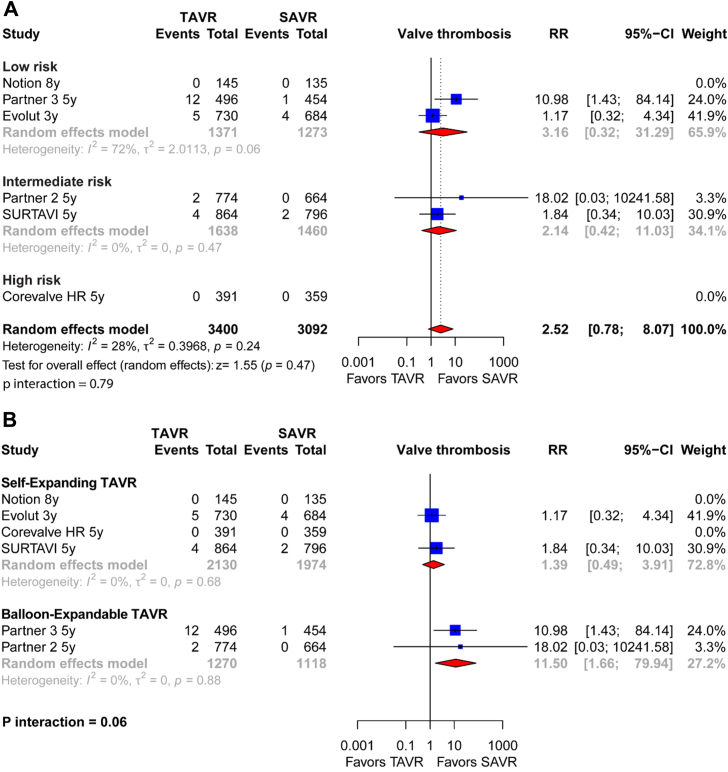
**Individual and summary risk ratios (RR) with their CI for valve thrombosis of studies comparing transcatheter aortic valve replacement (TAVR) vs surgical aortic valve replacement (SAVR).** Results are stratified by surgical risk (**A**) and TAVR valve type (**B**).

A total of 5 trials including 6492 patients reported thrombosis outcome. There was no significant difference in thrombosis risk between TAVR and SAVR (0.68% vs 0.23%: RR, 2.52 [0.78; 8.07]; *P* = .47). Heterogeneity was moderate (I^2^ = 28%). These results remained consistent across all risk profiles (*P* interaction = .79) (Figure 5A).

By the transcatheter valve stratified analysis, in comparison to SAVR, the valve thrombosis risk was lower with self-expanding (0.42%) than with balloon-expandable prostheses (1.1%) (*P* interaction = .06) (Figure 5B). Heterogeneity was null (I^2^ = 0%) in both groups.

The analysis of combined imaging features, which includes valve thrombosis, hypoattenuated leaflet thickening (HALT), and reduced leaflet motion, when stratified by percutaneous valve, showed results that were comparable to the analysis of thrombosis alone. In comparison to SAVR, there was a significantly higher risk of combined thrombosis risk in the balloon-expanding valve (4.41% vs 2.06%; RR, 2.17 [1.36; 3.48]; *P* = .001). However, this risk was not statistically significant in the self-expanding valves (2.63% vs 1.98%; RR, 1.34 [0.90; 2.00]; *P* = .14) (Supplemental Figure S5).

### Valve gradients

A total of 6 trials including 7505 patients contributed to the valve gradients outcome. Overall, 3840 of 7505 patients (51.2%) randomized to elective TAVR vs 3665 of 7505 patients (48.8%) randomized to SAVR had valve gradients, and their standard deviations were reported in the 2 arms.

**Figure 6:**
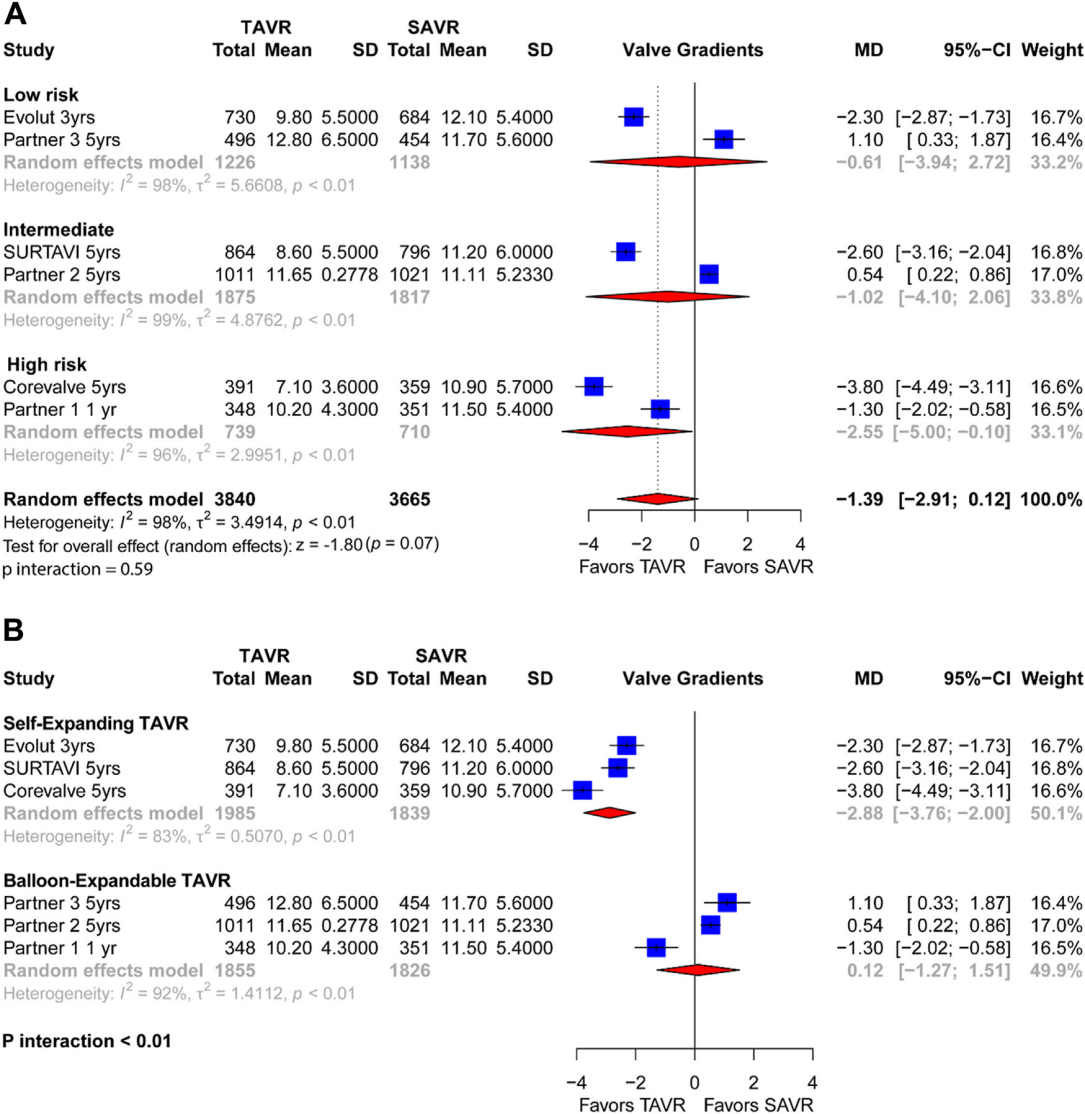
**Individual and summary mean differences (MD) with their standard deviations (SD) for valve gradients of studies comparing transcatheter aortic valve replacement (TAVR) vs surgical aortic valve replacement (SAVR).** Results are stratified by surgical risk (**A**) and TAVR valve type (**B**).

In the overall analysis, valve gradients were nominally lower in the TAVR vs SAVR cohort (MD −1.39 [−2.91 to 0.12]; *P* = .07) (Figure 6A). Heterogeneity was high (I^2^ = 98%).

No significant differences were observed in the effects on valve gradients when stratifying based on surgical risk profiles (*P* for interaction = .59) (Figure 6A). The high heterogeneity found in the overall analysis was explained by the distinct effect on gradient carried by the 2 TAVRs.

In the analysis by transcatheter valve type, we observed variability in valve gradients across groups. In comparison to SAVR, the self-expanding TAVR carried significantly lower valve gradients (MD −2.88 [−3.76 to −2.00]; *P* < .01) (Figure 6B). In contrast, for the balloon-expandable TAVR group, valve gradients were significantly increased, as compared to SAVR (MD 0.12 [−1.27 to 1.51]; *P* ≤ .01) (Figure 6B). Notably, the *P* value for interaction of <.01, derived from chi-square testing, indicates significant variability in the effect across different valve types, suggesting a subgroup-specific difference that warrants further exploration. Notably, the *P* value interaction between these subgroups was <.01, indicating statistical significance in the observed differences between them.

Four trials reported moderate-to-severe paravalvular leak at follow-up. Moderate-to-severe paravalvular leak occurred in 50/2099 (2.38%) in the TAVR vs 6/1960 (0.30%) in the SAVR group. Overall, moderate-to-severe leak risk was substantially higher in the TAVR group (HR, 5.42; 95% CI, 1.37-21.35; *P* = .01], without differences across risk groups (Supplemental Figure S6). No significant differences were observed in moderate-to-severe paravalvular leak stratified by valve type (Supplemental Figure S7).

## Discussion

The main results of the present large-scale meta-analysis of randomized trials encompassing 7785 patients at 5.76 ± 0.073 years follow-up can be summarized as follows as compared to SAVR:

1. Mortality or disabling stroke risk does not significantly differ between TAVR and SAVR. These results persisted in all 3 categories of estimated surgical risk (low, intermediate, and high). In the stratified analysis by transcatheter valve type, death or disabling stroke risk was lower with self-expanding (CoreValve/Evolut [Medtronic]) than with balloon-expandable valves (SAPIEN/SAPIEN 3 [Edwards Lifesciences]) (Central Illustration).

**Central Illustration:**
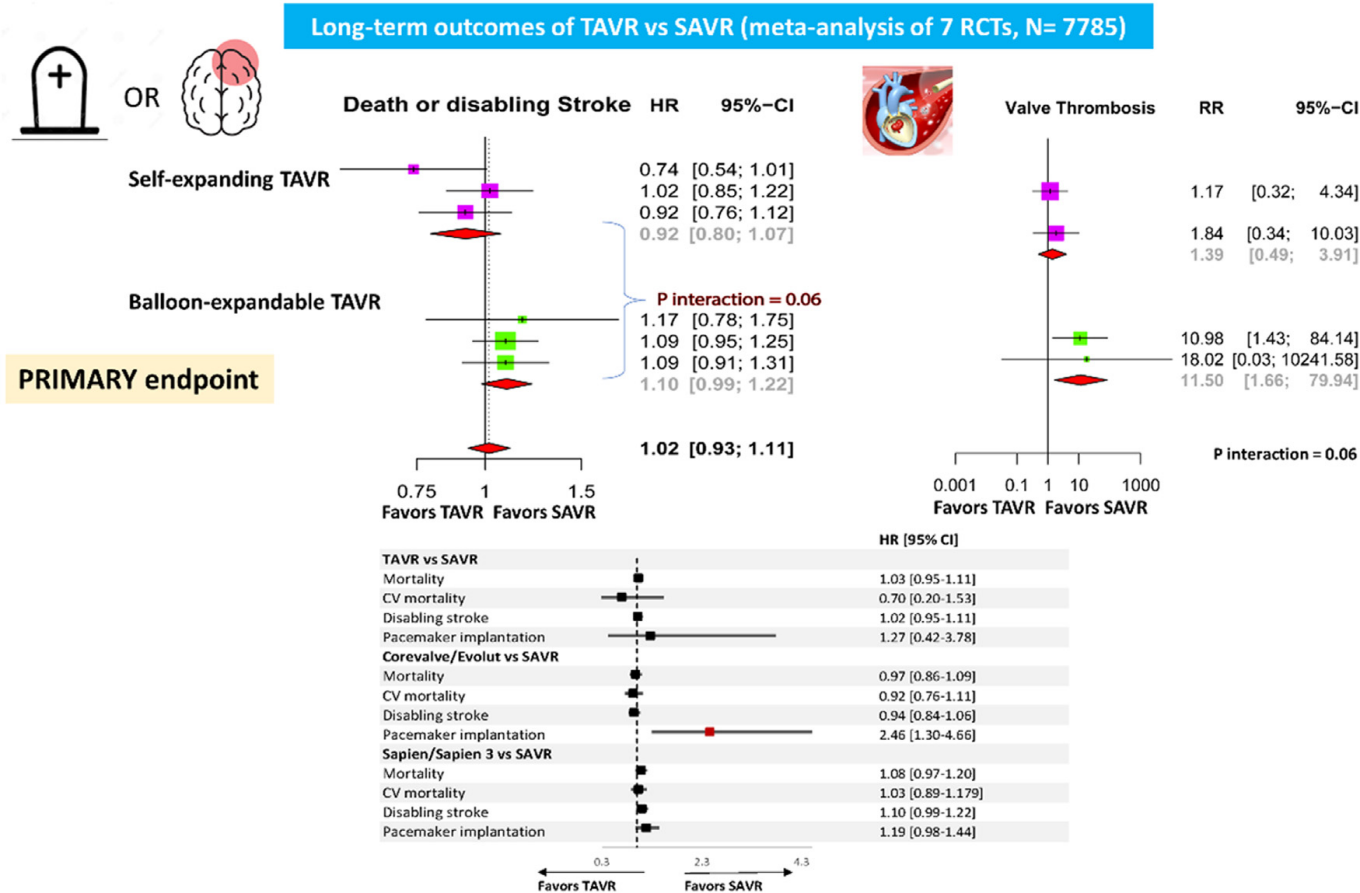
**Major findings on long-term effects of transcatheter aortic valve replacement (TAVR) vs surgical aortic valve replacement (SAVR).** Stratified analyses are applied by surgical risk and TAVR types. Long-term risk of mortality or disabling stroke are comparable between TAVR and SAVR (upper left); however, TAVR increases the likelihood of pacemaker implantation (bottom panel). Compared with SAVR, the relative reduction in death or stroke risk was greater with self-expanding than with balloon-expandable valves (upper panel) and may reflect the lower risk of valve thrombosis following self-expanding valves (top right panel). CV, cardiovascular; HR, hazard ratio; RCT, randomized controlled trials.
2. Mortality did not differ between TAVR and SAVR.
3. Self-expandable prostheses are associated with a significantly increased risk of pacemaker implantation, whereas balloon-expandable prostheses are not.
4. Thrombosis and valve gradients did not significantly differ between TAVR and SAVR. Self-expandable prostheses with supraannular implantation were associated with a significantly reduced risk for thrombosis and lower valve gradients than balloon-expandable prostheses with intraannular position.

To our knowledge, the current study is the largest-scale analysis at the longest available follow-up comparing TAVR and SAVR outcomes based on various risk profiles and types of transcatheter valves. This report enabled a thorough understanding of the relative effectiveness of TAVR and SAVR within different risk levels and specific percutaneous valve types.

Despite the rapid growth of TAVR, an emerging issue is valve thrombosis which must be systematically evaluated in the long-term.

An additional finding of the current report is that, as compared to SAVR, a lower thrombosis risk and lower mean valve gradients at the longest available follow-up were noted following self-expandable compared with balloon-expandable prostheses, although a direct comparison between percutaneous valves was not possible.

Leaflet thrombosis is not an inconsequential finding, as it may be associated with structural valve degeneration and increase the risk of cerebrovascular events.^22^

In our stratified analysis by transcatheter valve type, self-expanding valves are associated with a significantly lower risk of death or disabling stroke as compared with balloon-expandable valves. Of note, absence of heterogeneity (I^2^ = 0%) was found in both stratified meta-analyses for self-expanding and balloon-expandable devices, indicating consistency of the effect.

The method of valve deployment, the interaction between the prosthetic valve and the native aortic annulus, and the hemodynamic changes induced by the different types of valves might influence the thrombogenic risk. Accordingly, it has been demonstrated that the supraannular TAVR deployment results in nearly a 7-fold reduction in the size of the stagnation zone within the neo-sinus and a shorter blood residence time.^4^ Studies showed that intraannular valves have a slower washout time than supraannular valves.^23^, ^24^, ^25^

The definition of valve thrombosis was based on that reported in individual trials and did not account for HALT and reduced leaflet motion.

With the advent of more advanced imaging techniques, these features can now be assessed more accurately, suggesting that their impact may have been underestimated in earlier studies.^26^

Our additional analysis of combined imaging features, which includes valve thrombosis, HALT, and reduced leaflet motion, when stratified by percutaneous valve, strengthened our original estimates regarding thrombosis risk between percutaneous valves. However, further long-term data are required to confirm this observation. Consequently, the long-term magnitude and impact of valve thrombosis outcomes with different percutaneous valves merit further investigation. Another aspect related to valve gradient refers to the valve sizing. If the balloon-expandable valve is undersized or not perfectly matched to the patient’s anatomy, there might be a higher transvalvular gradient due to the suboptimal opening of the valve leaflets or flow obstruction. This hypothesis has been demonstrated in a real-world multicenter registry where a 4-dimensional multidetector computed tomography was performed after TAVR. It was found that early leaflet thrombosis was associated with a significantly higher mean transprosthetic gradient at discharge compared to patients without early leaflet thrombosis.^27^ Independent predictors of early leaflet thrombosis in balloon-expandable prostheses include low-flow, low-gradient AS, severe prosthesis-patient mismatch, and the use of a 29-mm prosthesis. However, no predictors were identified for early leaflet thrombosis in self-expandable prostheses.^27^

In our current study, a higher risk of pacemaker implantation was noted in patients receiving a TAVR in comparison with the surgical-treated individuals. This increased risk was more prominent in the lower surgical risk population and was driven by the self-expandable valve type. A potential reason for the higher pacemaker rates may be the implantation depth of self-expandable prosthesis.

Taken together, the findings of our meta-analysis show a good hemodynamic profile of the self-expandable prosthesis characterized by a low thrombosis risk and mild transprosthetic gradients but counterbalanced by a higher risk of pacemaker implants when compared to the balloon-expandable. These results highlight the importance of individualized patient selection for TAVR especially in the long-term and management strategies in the treatment of severe AS.

### Limitations

Trial-level data were included. However, consistency between the overall and the risk profile analyses support the reliability of the findings. Moreover, the estimates from individual trials, when categorized by percutaneous valve type, showed a consistent direction with low to no heterogeneity. The definition of valve thrombosis was based on that reported in individual trials. Thrombosis events were low across the studied groups (<1%), with the exception of balloon-expandable valves, which prevented a conclusive determination regarding this outcome. Conversely, the combined analysis of imaging features, which showed increased event rates between groups (ranging from 2% to 4%), confirmed the findings related to thrombosis alone. Additionally, the power calculations performed for the primary end point, which included death or disabling stroke, achieved a power exceeding 95%, thus confirming the robustness of the result. There was overall significant heterogeneity for the pacemaker and valve gradients (I^2^ > 90%) which should be taken into account when interpreting results for these outcomes. Accordingly, this heterogeneity was explained by the prespecified analyses conducted by transcatheter valve type which showed directionally opposite results for the self-expandable and balloon-expanding valves.

## Conclusions

In patients with severe AS, TAVR does not differ significantly from SAVR in terms of long-term risk of mortality or disabling stroke, across various surgical risk groups. However, TAVR increased the likelihood of requiring a pacemaker. Among TAVR valves, self-expanding models show a lower risk of death or stroke and valve thrombosis, but a higher pacemaker implantation rate compared to balloon-expandable valves. These findings support the comparable long-term safety and efficacy of TAVR, with specific considerations for valve type selection which may affect long-term valve durability as well as pacemaker requirements.
