# Supplementary material for: Long-Term Outcomes of Transcatheter vs Surgical Aortic Valve Replacement: Meta-analysis of Randomized Trials
Source: J Soc Cardiovasc Angiogr Interv. 2024 May 15;3(7):102143. doi: 10.1016/j.jscai.2024.102143 (PMC11307397; doi:10.1016/j.jscai.2024.102143)

**Supplementary Appendix**

Supplement to: Long-term outcomes of Transcatheter Aortic Valve vs Surgical Aortic Valve Replacement: meta-analysis of randomized trials

Contents

[Supplemental Table S1. Electronic search in Medline. 3](#_Toc165712888)

[Supplemental Table S2. Characteristics of included trials. 4](#_Toc165712889)

[Supplemental Table S3. Study risk of bias. 7](#_Toc165712890)

[Supplemental Table S4. Definition of valve thrombosis for each trials 8](#_Toc165712891)

[Supplemental Table S5. Egger’s test for the main outcomes 9](#_Toc165712892)

[Supplemental Figure S1. Cardiac mortality with TAVR vs SAVR. 10](#_Toc165712893)

[Supplemental Figure S2. Cardiac mortality with TAVR vs SAVR stratified by transcatheter valve type. 10](#_Toc165712894)

[Supplemental Figure S3. Stroke with TAVR vs SAVR. 12](#_Toc165712895)

[Supplemental Figure S4. Stroke with TAVR vs SAVR stratified by transcatheter valve type. 13](#_Toc165712896)

[Supplemental Figure S5. Combined thrombosis risk with self-expandable and balloon-expanding TAVR vs SAVR. 14](#_Toc165712897)

[
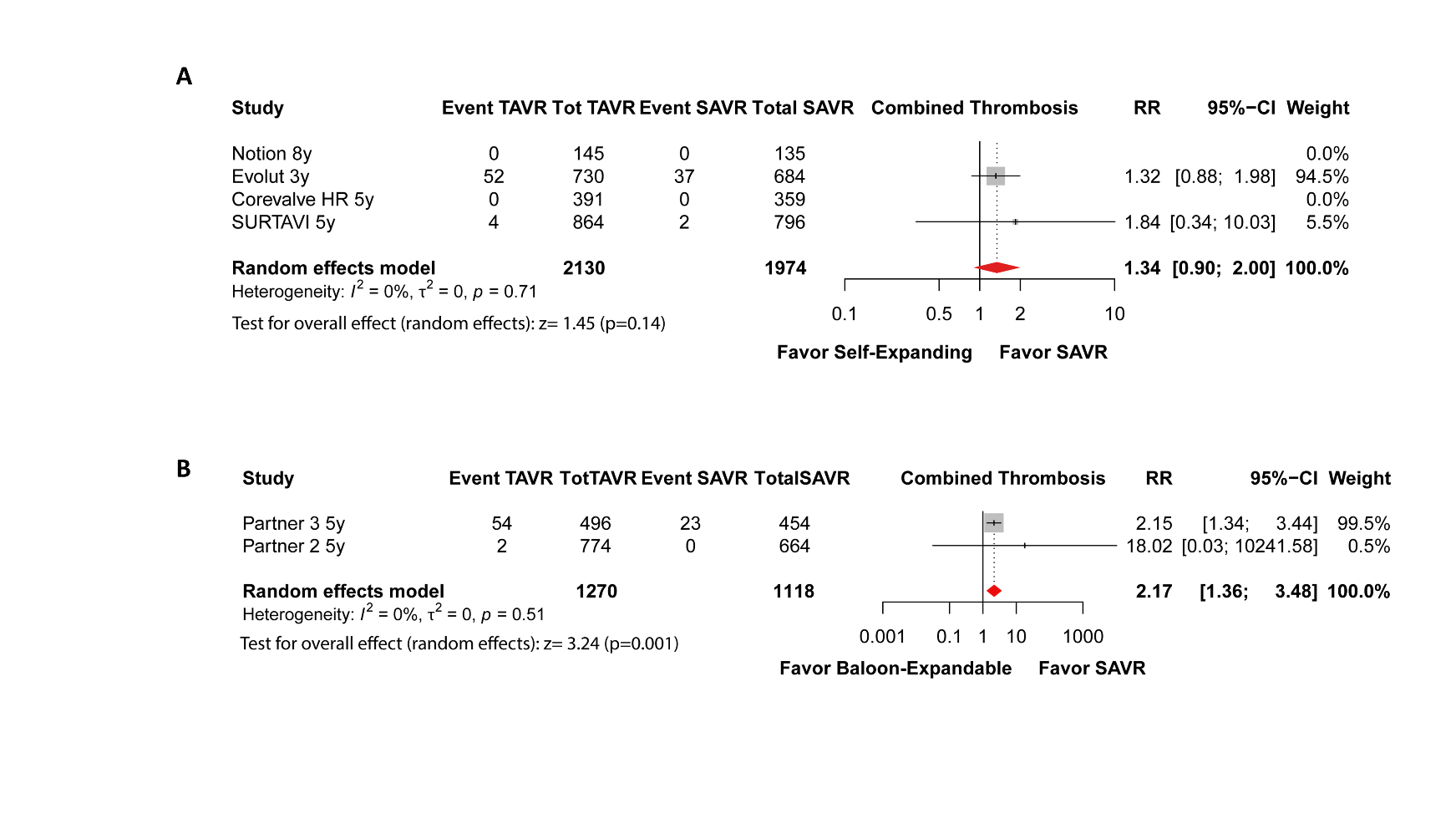
 14](#_Toc165712898)

[Supplemental Figure S6. Paravalvular leak with TAVR vs SAVR. 15](#_Toc165712899)

[Supplemental Figure S7. Paravalvular leak with TAVR vs SAVR stratified by transcatheter valve type. 16](#_Toc165712900)

# **Supplemental Table S1.** Electronic search in Medline.

| **Search** | **Query** | **Items found** |
| --- | --- | --- |
| #1 | Search: **((aortic valve disease) AND medical therapy and intervention)** Filters: **Randomized control Trial AND Clinical Trial** | 641 |
| #2 | Search: **((aortic valve disease) AND medical therapy)** Filters: **Randomized control Trial AND Clinical Trial** | 714 |
| #3 | Search: **((aortic valve disease) AND intervention)** Filters: **Randomized control Trial AND Clinical Trial** | 1572 |
| #4 | Search: **((severe aortic stenosis) AND medical therapy and intervention)** Filters: **Randomized control Trial AND Clinical Trial** | 308 |
| #5 | Search: **((severe aortic stenosis) AND medical)** Filters: **Clinical Randomized control Trial AND Clinical Trial** | 325 |
| #6 | Search: **((severe aortic stenosis) AND intervention)** Filters: **Randomized control Trial AND Clinical Trial** | 694 |
| #7 | Search: **((aortic valve) AND medical therapy and intervention)** Filters: **Randomized control Trial AND Clinical Trial** | 782 |
| #8 | Search: **((aortic valve) AND medical therapy)** Filters: **Randomized control Trial AND Clinical Trial** | 869 |
| #9 | Search: **((aortic valve) AND intervention)** Filters: **Randomized control Trial AND Clinical Trial** | 1982 |
| #10 | Search: **(TAVR)** Filters: **Randomized control Trial AND Clinical Trial** | 298 |
| #11 | Search: **(SAVR)** Filters: **Randomized control Trial AND Clinical Trial** | 101 |

# Supplemental Table S2. Characteristics of included trials.

| **Study** | **Key qualifying event** | **Number of participants** | **Strategy** | **Surgical Risk** | **Valve Type** | **Years of follow-up** | **Main age at baseline** |
| --- | --- | --- | --- | --- | --- | --- | --- |
| Notion | Clinical outcomes including, all-cause mortality, cardiovascular mortality, stroke, and permanent pacemaker implantation in patients with severe aortic valve stenosis undergoing SAVR or TAVR. | 280 | TAVR vs SAVR | Low | Self-expanding | up to 10 | 79.1 |
| Evolut | Clinical outcomes including death and disabling stroke, all-cause mortality, cardiovascular mortality, stroke, and permanent pacemaker implantation. Valve durability assessed based on aortic gradient and paravalvular leak in patients with severe aortic valve stenosis undergoing SAVR or TAVR. | 1414 | TAVR vs SAVR | Low | Self-expanding | up to 4 | 74 |
| Core Valve | Clinical outcomes including death and disabling stroke, all-cause mortality, cardiovascular mortality, stroke, and permanent pacemaker implantation Valve durability assessed based on aortic gradient and paravalvular leak in patients with severe aortic valve stenosis undergoing SAVR or TAVR. | 750 | TAVR vs SAVR | High | Self-expanding | 5 | 83 |
| SURTAVI | Clinical outcomes including death, disabling stroke, all-cause mortality, stroke, and permanent pacemaker implantation. Valve durability assessed based on aortic gradient and paravalvular leak in patients with severe aortic valve stenosis undergoing SAVR or TAVR. | 1660 | TAVR vs SAVR | Intermediate | Self-expanding | 5 | 79.8 |
| Partner 1 | Clinical outcomes including death and disabling stroke, all-cause mortality, cardiovascular mortality, stroke, and permanent pacemaker implantation. Valve durability assessed based on aortic gradient in patients with severe aortic valve stenosis undergoing SAVR or TAVR. | 699 | TAVR vs SAVR | High | Balloon-expandable | 5 | 83 |
| Partner 2 | Clinical outcomes including death and disabling stroke, all-cause mortality, cardiovascular mortality, stroke, and permanent pacemaker implantation. Valve durability assessed based on aortic gradient and paravalvular leak in patients with severe aortic valve stenosis undergoing SAVR or TAVR. | 2032 | TAVR vs SAVR | Intermediate | Balloon-expandable | 5 | 81.6 |
| Partner 3 | Clinical outcomes including death and disabling stroke, all-cause mortality, cardiovascular mortality, stroke, and permanent pacemaker implantation. Valve durability assessed based on aortic gradient and paravalvular leak in patients with severe aortic valve stenosis undergoing SAVR or TAVR. | 950 | TAVR vs SAVR | Low | Balloon-expandable | 5 | 73 |

# **Supplemental Table S3.** Study risk of bias.

# Supplemental Table S4. Definition of valve thrombosis for each trials

| **Studies** | **Definition of valve thrombosis** |
| --- | --- |
| CoreValve | Any thrombus not caused by infection attached to or near the trial valve that occludes part of the blood flow path, interferes with valve function, or is sufficiently large to warrant treatment. Valve thrombus found at autopsy in a subject whose cause of death was not valve-related or found at operation for an unrelated indication should also be counted as valve thrombosis. |
| Evolut Low Risk | Clinical valve thrombosis was defined as any thrombus not caused by infection attached to or near the implanted valve that occludes part of the blood flow path, interferes with valve function, or is sufficiently large to warrant treatment and is associated with any of the following clinical sequelae: any ischemic stroke, any peripheral embolic event, ST-segment elevation or non–ST-segment elevation myocardial infarction, or hemodynamic impairment associated with worsening heart failure. Subclinical valve thromboses were defined as those without evidence of clinical sequelae causing a hemodynamic impediment meeting the following criteria: an increase in aortic regurgitation resulting in moderate or severe, postdischarge mean gradient of ≥20 mm Hg that increased by >50%, or a decrease in the Doppler velocity index by >50% from discharge. |
| Notion | Thrombus development on any structure of the prosthetic valve leading to dysfunction. |
| SURTAVI | Any thrombus not caused by infection attached to or near the trial valve that occludes part of the blood flow path interferes with valve function or is sufficiently large to warrant treatment. Valve thrombus found at autopsy in a subject whose cause of death was not valve-related or found at operation for an unrelated indication should also be counted as valve thrombosis. |
| PARTNER 2 | Any thrombus attached to or near an implanted valve that occludes part of the blood flow path, interferes with valve function, or is sufficiently large to warrant treatment. Note that valve-associated thrombus identified at autopsy in a patient whose cause of death was not valve-related should not be reported as valve thrombosis. |
| PARTNER 3 | According to the definitions VARC-2 and VARC-3:   - VARC-2; Any thrombus attached to or near an implanted valve that occludes part of the blood flow path, interferes with valve function, or is sufficiently large to warrant treatment. Note that valve-associated thrombus identified at autopsy in a patient whose cause of death was not valve-related should not be reported as valve thrombosis. - VARC-3; Clinically significant valve thrombosis. Clinical sequelae of a thromboembolic event (e.g. stroke, TIA, retinal occlusion, other evidence of systemic thromboembolism) or worsening valve stenosis/regurgitation (e.g. signs of heart failure, syncope) and Hemodynamic valve deterioration Stage 2 or 3 or confirmatory imaging (CT evidence of HALT or TEE findings). In the absence of clinical sequelae, both hemodynamic valve deterioration Stage 3 and confirmatory imaging (CT evidence of HALT or TEE findings). |

# Supplemental Table S5. Egger’s test for the main outcomes

|  | **Intercept** | **P value** |
| --- | --- | --- |
| Death or disabling stroke | -0.57 | 0.81 |
| Death | -0.82 | 0.53 |
| Valve thrombosis | -10.46 | 0.21 |
| Pacemaker implantation | 2.22 | 0.08 |
| Moderate-to-severe parvalvular regurgitation | -2.83 | 0.51 |

# Supplemental Figure S1. Cardiac mortality with TAVR vs SAVR.


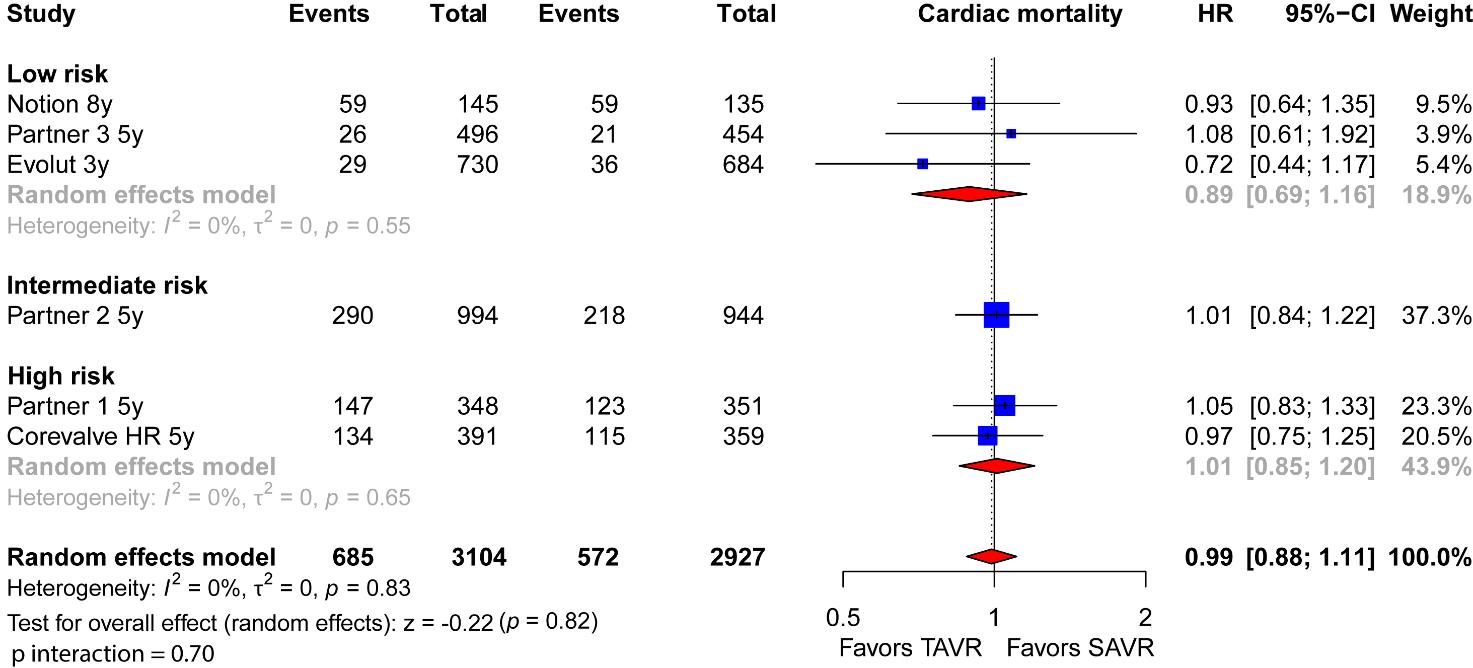


# Supplemental Figure S2. Cardiac mortality with TAVR vs SAVR stratified by transcatheter valve type.


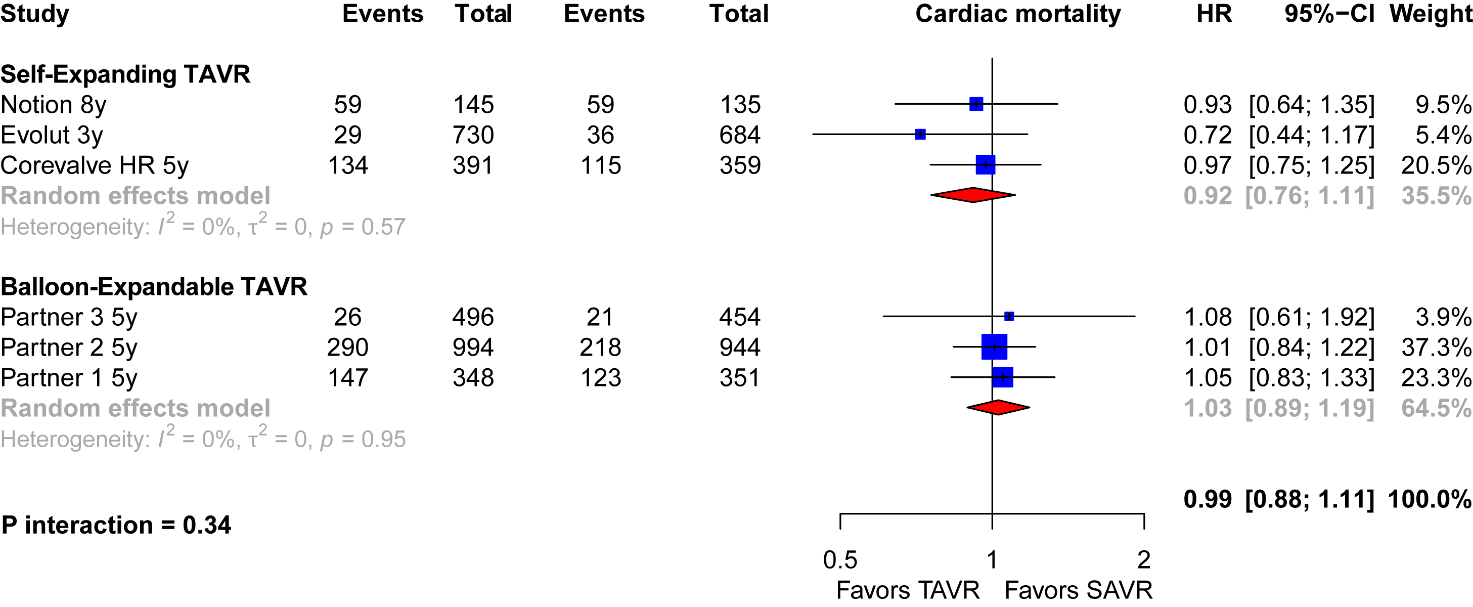


# Supplemental Figure S3. Stroke with TAVR vs SAVR.

**
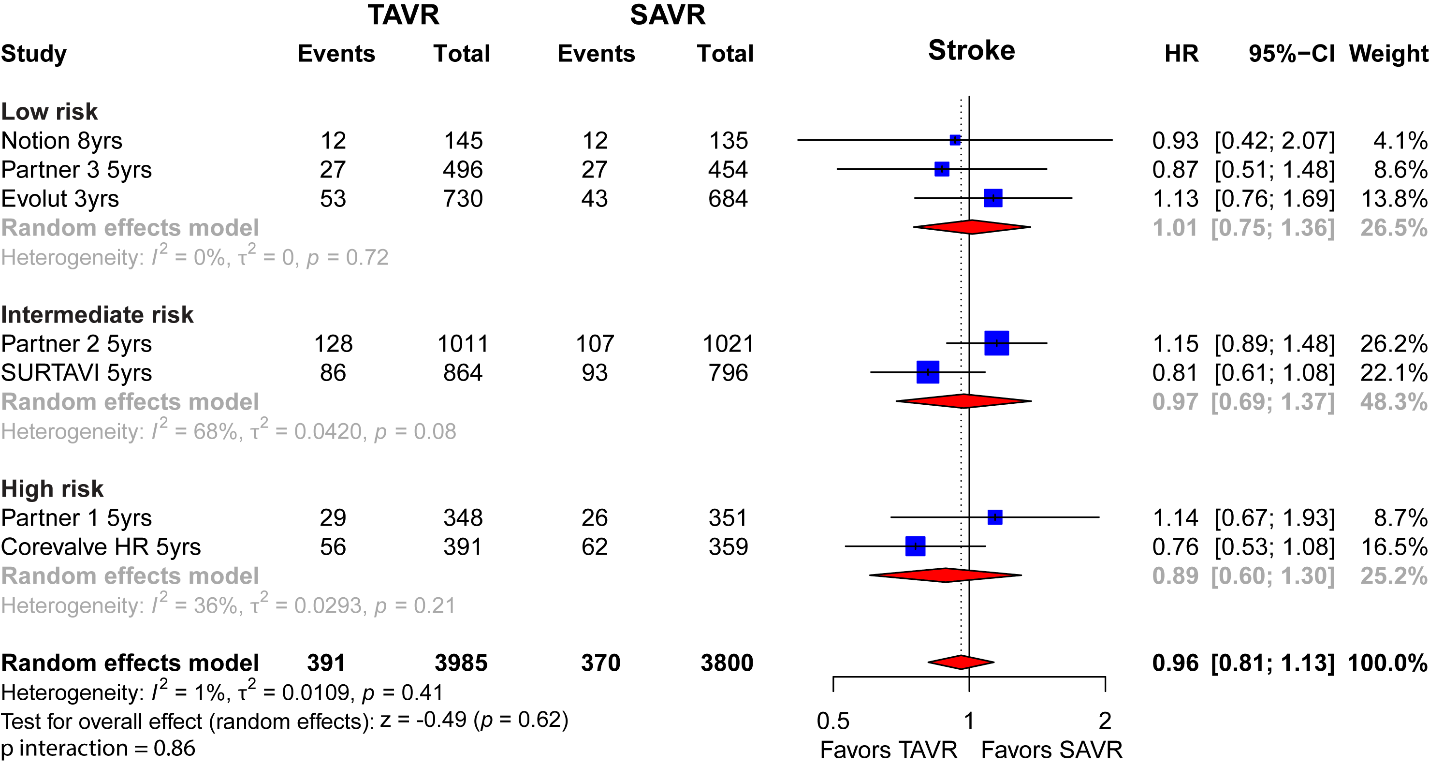
**

# Supplemental Figure S4. Stroke with TAVR vs SAVR stratified by transcatheter valve type.

**
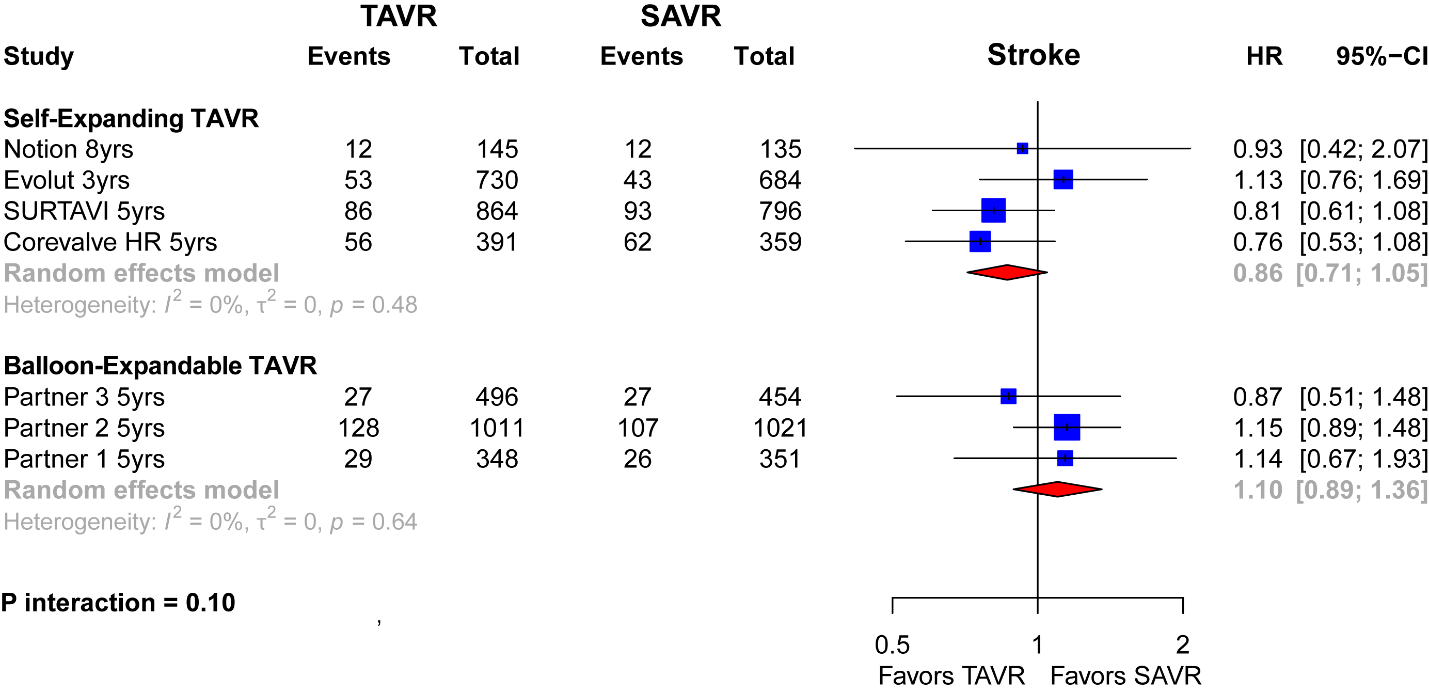
**

# Supplemental Figure S5. Combined thrombosis risk with self-expandable and balloon-expanding TAVR vs SAVR.

#
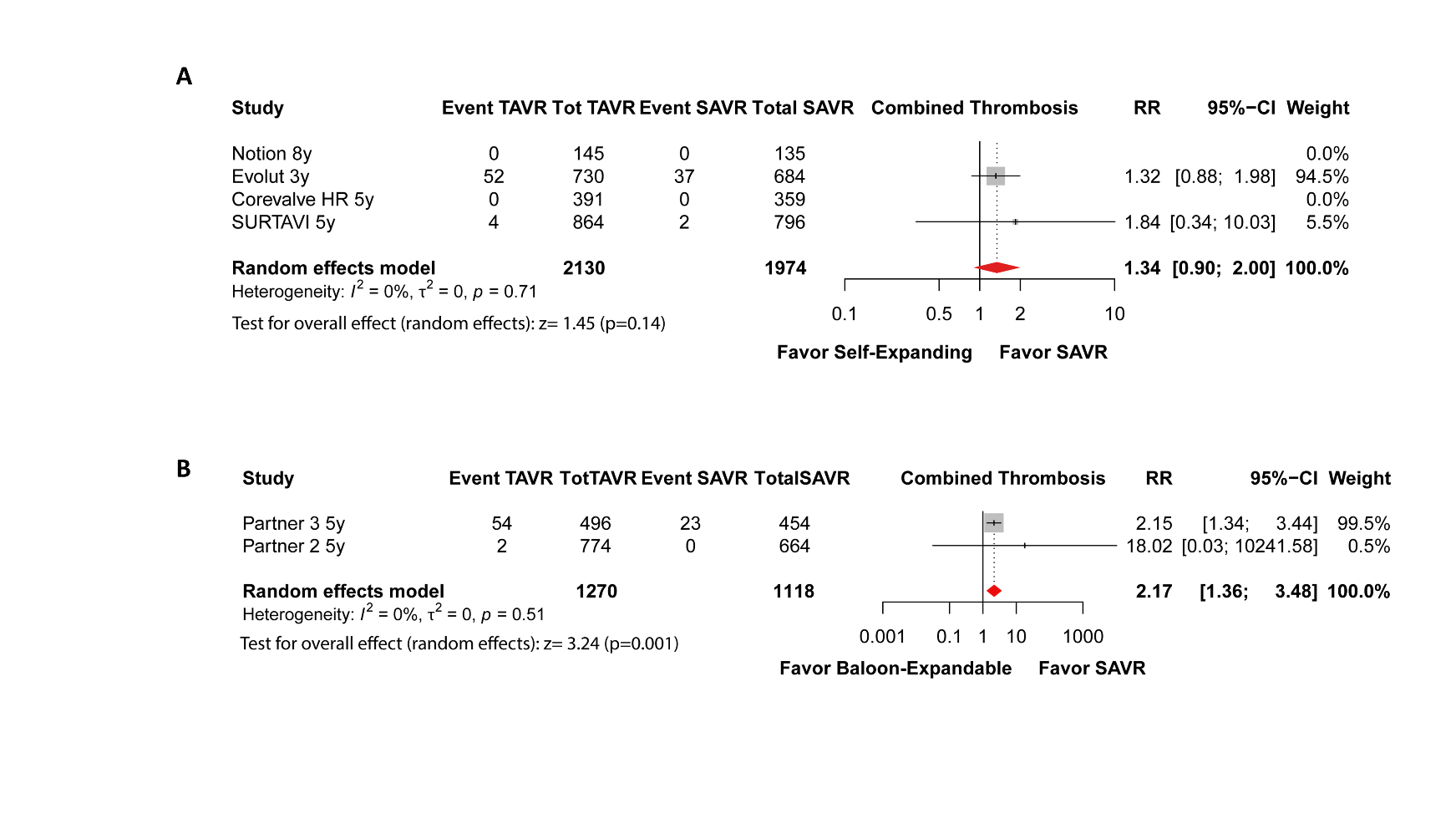


# Supplemental Figure S6. Paravalvular leak with TAVR vs SAVR.


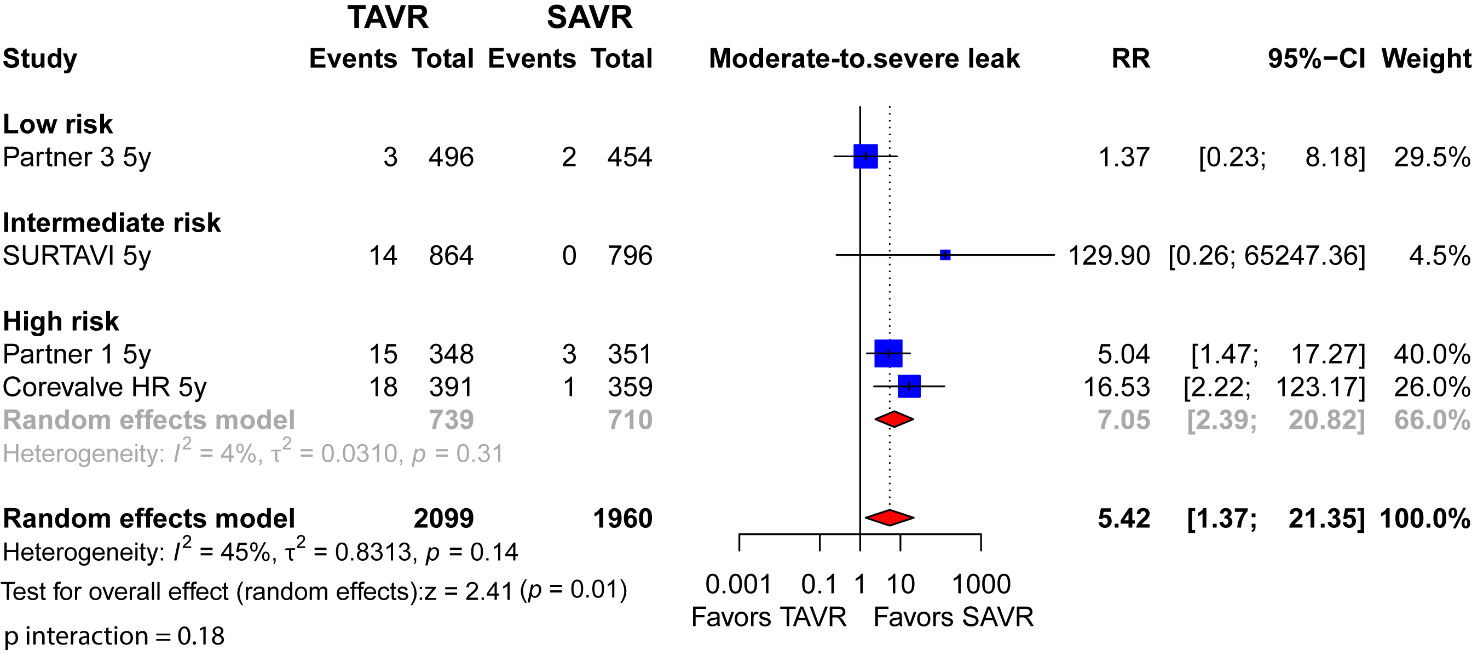


# Supplemental Figure S7. Paravalvular leak with TAVR vs SAVR stratified by transcatheter valve type.


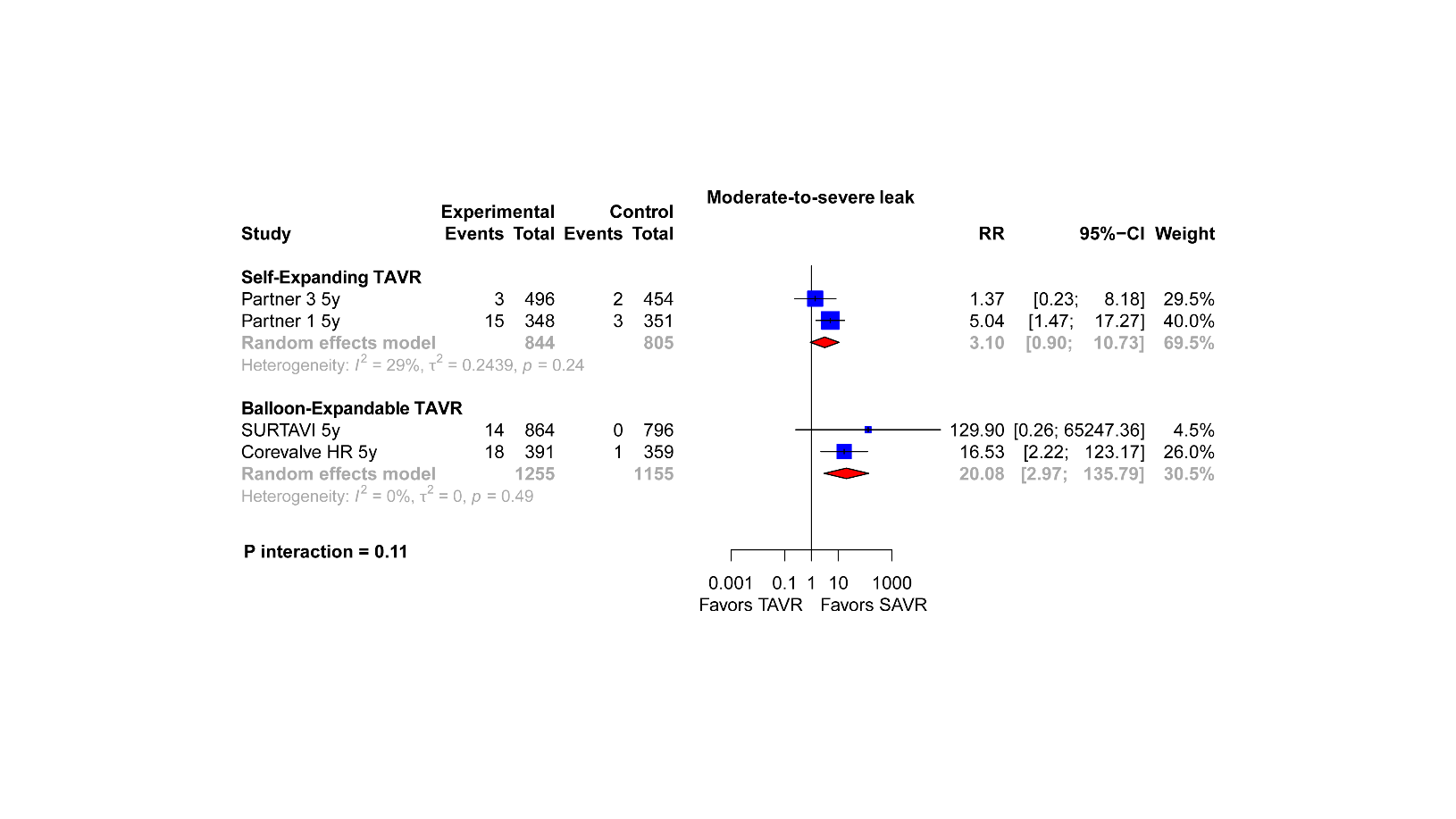

Supplement: Supplemental Tables S1-S5 and Supplemental Figures S1-S7 [file mmc1.docx]
